# Preclinical Evidence and Possible Mechanisms of *Rhodiola rosea L*. and Its Components for Ischemic Stroke: A Systematic Review and Meta-Analysis

**DOI:** 10.3389/fphar.2021.736198

**Published:** 2021-11-05

**Authors:** Yan Li, Miao Cai, Gen-Xiang Mao, Qin-Fen Shu, Xiao-Bei Liu, Xiao-Li Liu

**Affiliations:** ^1^ Department of Neurology, Zhejiang Hospital, Hangzhou, China; ^2^ Zhejiang Provincial Key Lab of Geriatrics and Geriatrics Institute of Zhejiang Province, Department of Geriatrics, Zhejiang Hospital, Hangzhou, China; ^3^ Department of Neurology, The No.1 People’s Hospital of Pinghu, Jiaxing, China

**Keywords:** Rhodiola rosea L, traditional medicine, ischemia stroke, possible mechanisms, preclinical evidence

## Abstract

**Background:** *Rhodiola rosea L*. has long been used as traditional medicines in Europe and Asia to treat a variety of common conditions and diseases including Alzheimer’s disease, cardiovascular disease, cognitive dysfunctions, cancer, and stroke. Previous studies reported that *Rhodiola rosea L*. and its components (RRC) improve ischemia stroke in animal models. Here, we conducted a systematic review and meta-analysis for preclinical studies to evaluate the effects of RRC and the probable neuroprotective mechanisms in ischemic stroke.

**Methods:** Studies of RRC on ischemic stroke animal models were searched in seven databases from inception to Oct 2021. The primary measured outcomes included the neural functional deficit score (NFS), infarct volume (IV), brain water content, cell viability, apoptotic cells, terminal deoxynucleotidyl transferase (TdT)-mediated dUTP-biotin nick end labeling (TUNEL)-positive cells, B-cell lymphoma-2 (Bcl-2) level and tumor necrosis factor-α (TNF-α) level. The secondary outcome measures were possible mechanisms of RRC for ischemic stroke. All the data were analyzed via RevMan version 5.3.

**Results:** 15 studies involving 345 animals were identified. Methodological quality for each included studies was accessed according to the CAMARADES 10-item checklist. The quality score of studies range from 1 to 7, and the median was 5.53. Pooled preclinical data showed that compared with the controls, RRC could improve NFS (Zea Longa (*p* < 0.01), modified neurological severity score (mNSS) (*p* < 0.01), rotarod tests (*p* < 0.01), IV (*p* < 0.01), as well as brain edema (*p* < 0.01). It also can increase cell viability (*p* < 0.01), Bcl-2 level (*p* < 0.01) and reduce TNF-α level (*p* < 0.01), TUNEL-positive cells (*p* < 0.01), apoptotic cells (*p* < 0.01).

**Conclusion:** The findings suggested that RRC can improve ischemia stroke. The possible mechanisms of RRC are largely through antioxidant, anti-apoptosis activities, anti-inflammatory, repressing lipid peroxidation, antigliosis, and alleviating the pathological blood brain barrier damage.

## Introduction

Ischemic stroke, a common neurological disease, has been the major cause for the central nervous system dysfunction with a relative high mortality and morbidity in clinical practice (Benjamin et al., 2017; Benjamin et al., 2018). The burden of stroke will increase greatly during the next 20 years because of the aging population, especially in developing countries (Donnan et al., 2018). Cerebral ischemia causes several pathological processes, such as inflammation, oxidative stress, cell apoptosis, ion imbalance, and calcium overload (Jayaraj et al., 2019) leading to neurologic deficits in ischemic stroke. Unfortunately, intravenously recombinant tissue plasminogen activator (rtPA) is so far the only Food and Drug Administration (FDA)-approved thrombolytic agent for treating ischemia stroke within the golden hour 4.5 h of stroke onset (National Institute of Neurological Disorders and Stroke rt-PA Stroke Study Group., 1995; Sandercock et al., 2012). Due to the narrow therapeutic window, several contraindications and the incidence of hemorrhagic transformation, rtPA remains largely underutilized (Medcalf, 2011). Moreover cerebral ischemia/reperfusion injuries can also lead to severe adverse reactions (Jickling et al., 2014). In spite of the substantial research and development efforts, the available therapeutic options remains insufficient for acute ischemic stroke. Owing to the limitations of the current available treatments, complementary and/or alternative medicine is thus increasingly sought to treat stroke worldwide.

*Rhodiola rosea L.* also named Rhodiola, Golden Root, Arctic Root, and Roseroot, belongs to the plant family of Crassulaceae and genus Rhodiola (Khanum et al., 2005), and is widely distributed in Asia, Europe and North America (Elameen et al., 2020). In traditional Russian (Siberian) folk medicine, *Rhodiola rosea L.* has been used as an adaptogenic medicinal product for a long time (Ioset et al., 2011), and the plant is useful for increasing mental and physical capacities (Panossian., et al., 2021). Modern pharmacological researchies have revealed multiple bioactivities from *Rhodiola rosea L.* and its components (RRC) such as anti-oxidative (Zhang et al., 2007), anti-inflammation (Pu et al., 2020), anti-fatigue (Shevtsov et al., 2003), immune enhancement (Tao et al., 2019) and neuro-protective effects (Yu et al., 2008), for treatment of Alzheimer’s disease, cardiovascular disease, cognitive dysfunctions, cancer, and stroke (Zhong et al., 2019; Fan et al., 2020). Salidroside, rhodiosin, p-tryosol, pyridrde, rosavin, rhodionin (Zhang et al., 2006) and ferulic acid eicosyl ester (Michels et al., 2018) are the main bioactive compounds in the Rhodiola species.

An objective and quantitative systematic review of preclinical studies is a type of secondary research, may identify confounding factors across animal studies (Ritskes-Hoitinga et al., 2014). Systematic reviews are a powerful approach to offer credible evidence and be favourable for selecting the appropriate drug administration for future clinical trials (van Luijk et al., 2013). However, the current evidence of RRC for ischemic stroke still lack systematic analysis. Therefore, in the present study we conduct a preclinical systematic review of RRC on ischemia stroke to further reveal the basis of action and the neurochemical modulatory mechanism of RRC in animal model of ischemia stroke.

## Methods

### Search Strategy

A comprehensive search was performed to identify experimental studies evaluating the effects of RRC for ischemia stroke from databases, including PubMed, embase, CBM, Web of Science, National Knowledge Infrastructure (CNKI), Wanfangdatabase and VIP information database. All searches were electronically searched from the inception up to Oct 2021. Studies about assessing the effectiveness of RRC for ischemic stroke in animals were identified. Our literature search strategy was as following: (Rhodiola OR Rhodiola rosea OR Roseroot OR Rhodioloside OR Salidroside) AND (Ischemic stroke OR Cerebral ischemic injury OR Cerebral infarction OR Brain infraction).

### Eligibility Criteria

Experimental studies evaluating the effect of RRC for ischemic stroke were selected. Two authors independently screened the titles and/or abstracts according to the search strategy. Then, we assessed the full-text articles for eligibility. Studies were included if they met the following criteria: 1) Animal models were established for ischemic stroke; 2) RRC as monotherapy was administrated in the experimental group, regardless of its mode, dosage, and frequency. 3) The primary measured outcomes were neural functional deficit score (NFS), infarct volume (IV), brain water content, cell viability, apoptotic cells, terminal deoxynucleotidyl transferase (TdT)-mediated dUTP-biotin nick end labeling (TUNEL)-positive cells, B-cell lymphoma-2 (Bcl-2) level and tumor necrosis factor-α (TNF-α) level. The secondary outcome measures were mechanisms of RRC for ischemic stroke; and 4) The control group received vehicle or no adjunct intervention.

### Exclusion Criteria

The prespecified exclusion criteria were as follows: 1) the targeting disease was not ischemic stroke; 2) RRC were used as combination; 3) the article was a clinical or *in vitro* study; 4) the study was a case report, clinical trial, review, abstract, comment, editorial, duplicate publication or *in vitro* study, and 5) lack of the control group.

### Data Extraction

Two independent reviewers assessed the articles and the following details were extracted: 1) the first author, publication year; 2) individual data from each study, including animal species, gender, samples for individual comparison, and weight; 3) type of animal model; 4) type of anesthetic; 5) intervention characteristics from both treatment and control groups, including drug, timing for initial treatment, dosage, mode, and frequency; 6) outcome measures and its corresponding pvalue. For each comparison, the mean value and standard deviation from each treatment and control group of every study were extracted. If the data were demonstrated graphically, we tried to contact the author for further information or digital ruler software was applied. Otherwise we only performed qualitative analysis. The data of highest dose was selected when the treatment group included various doses of the target drug. The result of the last time point was included when the data were expressed at different times.

### Quality Assessment

Two authors independently assessed the methodological quality of the included articles according to the Collaborative Approach to Meta-Analysis and Review of Animal Data from Experimental Studies (CAMARADES) 10-item checklist (Sena et al., 2007): 1) peer-reviewed publication; 2) statements of temperature control; 3) randomization to treatment or control group; 4) blinded induction of model; 5) blinded assessment of outcome; 6) use of anesthetic without significant intrinsic neuroprotective activity; 7) appropriate animal model; 8) sample size calculation; 9) compliance with animal welfare regulations; and 10) declaration of potential conflict of interests. Each study was given an aggregate quality score based on one-point awarding for each item. Discrepancies were resolved by discussion or consultation with corresponding author.

### Statistical Analysis

The pooled analyses were performed using RevMan 5.3 software. All outcome measures were considered as continuous data. To estimate the effect of RRC on ischemic stroke, the random effects model and standard mean difference (SMD) with 95% confidence intervals (CIs) were calculated. Heterogeneity among individual studies was assessed via *I* ^*2*^ statistics test. If probability value was less than 0.05, the difference was considered statistically significant.

## Results

### Study Inclusion

**FIGURE 1:**
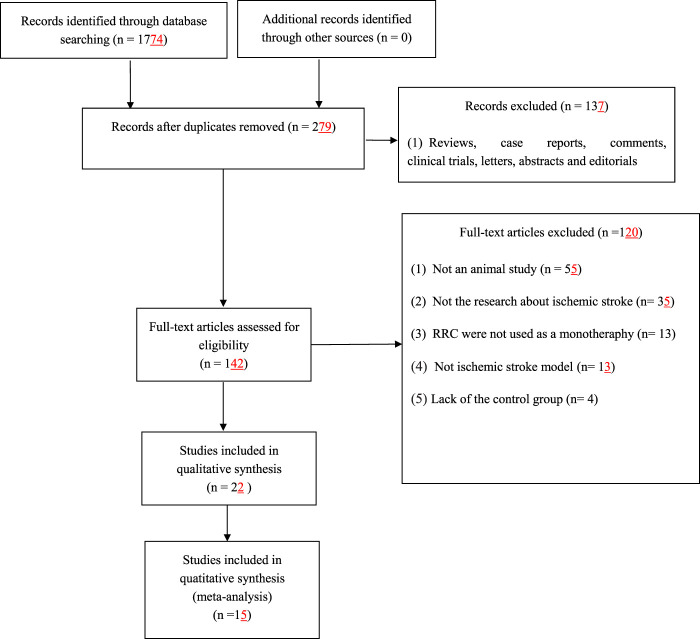
Flow diagram.

We identified 1774 potentially relevant articles from seven databases. After removal of duplicates and irrelevant articles, 279 records remained. By reviewing titles and abstracts, 137 studies were excluded because they were case reports, abstracts, comments, clinical trials, editorials, letters and review articles. After going through the remaining full-text articles, 120 articles were excluded for at least one of following reasons: 1) the article was not a research about ischemic stroke; 2) not an *in vivo* study; 3) the intervention was a combination of RRC with potential effect on ischemic stroke; 4) the study did not access the effects of RRC on the animal model of ischemic stroke; 5) no control group. Finally, 15 eligible studies (Chen et al., 2009; Shi et al., 2012; Chen et al., 2014; Yu et al., 2014; Han et al., 2015; Atochin et al., 2016; Chen et al., 2016; Liu et al., 2018; Zhang et al., 2018; Zuo et al., 2018; Zhang et al., 2019; Zhong et al., 2019; Li et al., 2020; Zhang et al., 2020; Dong et al., 2021) involving 345 animals were identified **(** Figure 1).

### Characteristics of Included Studies

The basic characteristics of the eligible studies are summarized in Table 1. Fifteen studies included were published between 2009 and 2021. Among them, 13 studies were conducted in English and two studies (Chen et al., 2009; Chen et al., 2014) were Chinese paper. For animal species, 12 studies used rats including Sprague-Dawley (SD) rats (n = 144) and Wistar rats (n = 134) as animal models. Three studies used C57BL/6 mice (n = 67). The weight of rats ranged from 190 to 320 g, and the weight of mice ranged from 18 to 23 g. Cerebral ischemic injury in the included studies was induced by temporary middle cerebral artery occlusion (MCAO) in which ischemic time varied from 8 to 180 min (Chen et al., 2009; Shi et al., 2012; Chen et al., 2014; Yu et al., 2014; Han et al., 2015; Atochin et al., 2016; Liu et al., 2018; Zhang et al., 2018; Zuo et al., 2018; Zhang et al., 2019; Zhong et al., 2019; Li et al., 2020; Zhang et al., 2020; Dong et al., 2021), and permanent MCAO (Chen et al., 2016). For anesthesia chosen in experiments, four studies (Shi et al., 2012; Han et al., 2015; Atochin et al., 2016; Zhang et al., 2020) used chloral hydrate, three studies (Zuo et al., 2018; Zhang et al., 2019; Zhong et al., 2019) used isoflurane, four studies (Chen et al., 2016; Zhang et al., 2018; Li et al., 2020; Dong et al., 2021) used sodium pentobarbital, and the remaining four studies (Chen et al., 2009; Chen et al., 2014; Yu et al., 2014; Liu et al., 2018) did not report it. Ten studies utilized a dose gradient of RRC: one study (Atochin et al., 2016) used 5, 10 and 20 mg kg^−1^ intravenously, one study (Chen et al., 2016) used 20, and 40 mg kg^−1^ orally, one study (Han et al., 2015) used 15, and 30 mg kg^−1^ intraperitoneally, one study (Liu et al., 2018) used 2.5, 5, 10 and 20 mg kg^−1^ intravenously, two studies (Zhang et al., 2018; Li et al., 2020) used 25, 50 and 100 mg kg^−1^ intraperitoneally, one study (Zhang et al., 2019) used 20, 50 and 100 mg kg^−1^ intraperitoneally, one study (Zhong et al., 2019) used 20, 40, and 80 mg kg^−1^ intraperitoneally, one study (Zuo et al., 2018) used 10, 20, and 40 mg kg^−1^ intraperitoneally, and one study (Zhang et al., 2020) used 12 and 48 mg kg^−1^ through gavage. Six studies (Shi et al., 2012; Chen et al., 2014; Atochin et al., 2016; Chen et al., 2016; Zuo et al., 2018; Zhong et al., 2019) administrated RRC before stroke; Seven studies (Shi et al., 2012; Yu et al., 2014; Chen et al., 2016; Liu et al., 2018; Zhang et al., 2018; Zhang et al., 2020; Dong et al., 2021) administrated RRC after stroke; and two studies (Han et al., 2015; Li et al., 2020) administrated RRC before and after stroke. In the control group, twelve studies applied same volume of normal saline, one study (Liu et al., 2018) applied phosphate-buffered saline (PBS), one study (Zhang et al., 2020) applied distilled water and the remaining one study (Chen et al., 2015) did not clearly mentioned.

**TABLE 1:** Basic characteristics of the included studies.

Study (years)	Species (sex, n = experimental/control group)	Weight	Random method	Model (method)	Anesthetic	Method of administration		Outcome index (time)	Intergroup differences
						Experimental group	Control group		
Atochin et al. (2016)	Wister rats (male, 36/42)	250–300 g	NR	MCAO (2 h)	Chloral hydrate	P-tyrosol (5, 10 and 20 mg/kg, iv); before MCAO; once daily for 5 days	Normal saline (same volume, iv); onset the experiment; once daily for 5 days	1) NFS (McGraw scale, 1, 3ays and 5 days)	1) *p* < 0.05
								2) Neurons counts	2) *p* < 0.001
								3) Conjugated content	3) *p* < 0.05
Chen et al. (2015)	SD rats (male, 18/18)	200–250 g	NR	MCAO (permanent)	Pentobarbital sodium	Salidroside (20 and 40 mg/kg, orally); before MCAO; once	NR	1) IV (TTC, 1 d)	1) *p* < 0.01
								2) Cerebral edema	2) *p* < 0.01
								3) Cell survival	3) *p* < 0.01
								4) TNF-α content	4) *p* < 0.05
								5) IL-1β content	5) *p* < 0.01
								6) IL-6 content	6) *p* < 0.05
								7) Bcl-2 expression	7) *p* < 0.01
								8) Bax expression	8) *p* < 0.01
Han et al. (2015)	SD rats (male, 7/7)	250–280 g	NR	MCAO (2 h)	Chloral hydrate	Salidroside (15 and 30 mg/kg, ip); once before MCAO and once after reperfusion	Normal saline (same volume, ip); once before MCAO and once after reperfusion	1) NFS (Zea Longa, 1 d)	1) *p* < 0.05
								2) IV (TTC, 1 d)	2) *p* < 0.05
								3) SOD, GST, GSH-Px activities	3) *p* < 0.05
								4) MDA content	4) *p* < 0.05
								5) Nrf2 and HO-1 expression	5) *p* < 0.05
Liu et al. (2018)	C57BL/6 mice (male, 12/12)	21–23 g	NR	MCAO (1 h)	NR	Salidroside (2.5, 5, 10 and 20 mg/kg, iv); after MCAO; once daily for 5 days	PBS (same volume, iv) after MCAO; once daily for 5 days	1) NFS (mNSS, 3 days)	1) *p* < 0.05
								2) NFS (Rotarod tests, 3, 5, 7, 9 and 11 days)	2) *p* < 0.001
								3) IV (TTC, 3 days)	3) *p* < 0.01
								4) Brain loss (HE)	4) *p* < 0.05
								5) IL-1β expression	5) *p* < 0.001
								6) IL-2 expressions	6) *p* < 0.01
								7) IL-6 expressions	7) *p* < 0.01
								8) IL-8 expression	8) *p* < 0.001
								9) TNFα expression	9) *p* < 0.001
								10) MBP expression	10) *p* < 0.01
								11) MAP2 expression	11) *p* < 0.01
Shi et al. (2012)	SD rats (male, 6/6)	280–300 g	NR	MCAO (2 h)	Chloral hydrate	Salidroside (12 mg/g, iv); before MCAO; once daily for 7 days	Normal saline; (same volume, iv); before the MCAO; once daily for 7 days	1) NFS (Zea Longa, 1 d)	1) *p* < 0.01
								2) IV (TTC, 1 d)	2) *p* < 0.01
								3) HE staining	3) *p* < 0.01
								4) Cell viability	4) *p* < 0.05
								5) Apoptotic cells	5) *p* < 0.05
								6) ROS level	6) *p* < 0.01
								7) Bcl-2 expression	7) *p* < 0.01
								8) Bax expression	8) *p* < 0.01
Zhang et al. (2018)	C57BL/6 mice (male, 8/8)	18–22 g	NR	MCAO (2 h)	Pentobarbital sodium	Salidroside (25, 50 or 100 mg/kg ip); after MCAO; once daily for 3 days	Normal saline; (same volume, ip); after MCAO; once daily for 3 days	1) NFS (Zea Longa, 1 d)	1) *p* < 0.05
								2) IV (TTC, 1 d)	2) *p* < 0.05
								3) Cell viability	3) *p* < 0.05
								4) Brain edema	4) *p* < 0.05
								5)TUNEL positive cells count	5) *p* < 0.05
								6) LDH activity	6) *p* < 0.01
								7) Apoptotic cells	(7) *p* < 0.01
								8) Bcl-2 expression	(8) *p* < 0.01
Zhang et al. (2019)	SD rats (male,6/6)	200–240 g	NR	MCAO (2 h)	Isoflurane	Salidroside (20, 50 and 100 mg/kg, ip.); after MCAO; once daily for 7 d	Normal saline; (same volume, ip); after MCAO; once daily for 7 d	1) NFS (Zea Longa, 7 days)	1) *p* < 0.01
								2) IV (MRI, 1 and 7 days)	2) *p* < 0.01
								3) NeuN protein level	3) *p* < 0.05
								4) TNFα mRNAs level	4) *p* < 0.05
								5) IL-6 mRNAs level	5) *p* < 0.05
Yu et al. (2014)	SD rats (male)	190–210 g	NR	MCAO (2 h)	NR	Salidroside (50 mg/g, iv); once after MCAO	Normal saline; (same volume, iv); once after MCAO	1) NFS (mNSS, 1 d)	1) *p* < 0.01
								2) IV (TTC, 1 d)	2) *p* < 0.01
								3) Cell viability	3) *p* < 0.01
								4) Apoptotic cells	4) *p* < 0.01
								5) TUNEL positive cells count	5) *p* < 0.01
								6) Glucose uptake	6) *p* < 0.01
								7) GLUT3 expression	7) *p* > 0.05
								8) pS133-CREB level	8) *p* > 0.05
								9) PKA RII level	9) *p* < 0.05
								10) Intracellular Ca2+ influx	10) *p* < 0.05
Zuo et al. (2018)	SD rats (male, 10 10/)	260–280 g	NR	MCAO (3 h)	Isoflurane	Salidroside (10, 20 and 40 mg/kg, ip); once before MCAO	Normal saline; (same volume, ip); once before MCAO	1) NFS (Ludmila Belayev, 1, 2, 3, 4, 5, 6 and 7 days)	1) *p* < 0.05
								2) NFS (For grid test, 1, 2, 3, 4, 5, 6 and 7 days)	2) *p* < 0.05
								3) NFS (Beam walk test, 1, 2, 3, 4, 5, 6 and 7 days)	3) *p* < 0.05
								4) NFS (Wire grip test, 1, 2, 3, 4, 5, 6 and 7 days)	4) *p* < 0.05
								5) IV (TTC, 1 d)	5) *p* < 0.05
								6) Evan’s blue leakage	6) *p* < 0.05
								7) Cell viability	7) *p* < 0.05
Chen et al. (2014)	Wister rats (male, 8/8)	220–250 g	NR	MCAO (2 h)	NR	Rhodiola rosea (0.672 g/kg, ig); after MCAO; once daily for 15 days	Normal saline; (same volume, ig); after MCAO; once daily for 15 days	1) NFS (Zea Longa, 24 h)	1) *p* < 0.01
								2) C-Fos expression	2) *p* < 0.01
								3) Apoptotic cells	3) *p* < 0.01
Chen et al. (2009)	Wister rats (male, 8/8)	280–320 g	NR	MCAO (2 h)	NR	Rhodiola rosea (0.672 g/kg, ig); before MCAO; once daily for 4 weeks	Normal saline; (same volume, ig); before MCAO; once daily for 4 weeks	1) NFS (Zea Longa, 3, 6, 24, 48 and 72 h)	1) *p* < 0.01
								2) TUNEL positive cells count	2) *p* < 0.05
								3) GFAP expression	3) *p* < 0.05
Zhong et al. (2019)	SD rats (male, 10/10)	270–290 g	NR	MCAO (2 h)	Isoflurane	Salidroside (20, 40 and 80 mg/kg, ip); once before MCAO	Normal saline; (same volume, ip); once before MCAO	1) NFS (mNSS, 24 h)	1) *p* < 0.01
								2) NFS (Balance beam test 24 h)	2) *p* < 0.01
								3) NFS (The foot fault test 24 h)	3) *p* < 0.001
								4) DA level	4) *p* < 0.01
								5) DOPAC level	5) *p* < 0.05
								6) HVA level	6) *p* < 0.05
								7) MAO level	7) *p* < 0.05
Zhang et al. (2020)	Wistar rats (male, 12/12)	250–300 g	NR	MCAO (8min)	Chloral hydrate	Salidroside (12 and 48 mg/kg, ig); after MCAO; once daily for 3 days	Distilled water; (same volume, ig); after MCAO; once daily for 3 days	1) NFS (6h, 1, 3, 5 and 7 days)	1) *p* < 0.05
								2) Apoptotic cells	2) *p* < 0.05
								3) P53 level	3) *p* < 0.05
								4) Bcl-2 level	4) *p* < 0.05
								5) Bax level	5) *p* < 0.05
Dong et al. (2021)	C57/BL6 mice (male, 10/17)	21–23 g	NR	MCAO (1 h)	Pentobarbital sodium	Salidroside (10 mg/g, iv); after MCAO; once daily for 14 days	Normal saline; (same volume, iv); after MCAO; once daily for 14 days	1) NFS (Rotarod tests, 1, 3, 5, 7, 9, 11,14, 17, 21 and 35 days)	1) *p* < 0.01
								2) GFAP level	2) *p* < 0.05
								3) cyclin D1 expression	3) *p* < 0.05
								4) CDK4 expression	4) *p* < 0.05
								5) p27Kip1 level	5) *p* < 0.05
Li et al. (2020)	SD rats (male, 15/15)	250–280 g	NR	MCAO (2 h)	Pentobarbital sodium	Salidroside (25, 50 and 100 mg/kg, ip); once before MCAO and once after reperfusion and then once daily for 7 days	Normal saline; (same volume, ip); once before MCAO and once after reperfusion and then once daily for 7 days	1) NFS (Zea Longa, 7 days)	1) *p* < 0.01
								2) IV (TTC, 7 days)	2) *p* < 0.01
								3) Cell viability	3) *p* < 0.01
								3) FGF2/FGFR1 expression	4) *p* < 0.01
								4) TNFα expression	5) *p* < 0.01
								5) IL-1β expression	6) *p* < 0.01
								6) IL-6 expression	7) *p* < 0.01
								7) c-caspase 3 level	8) *p* < 0.01
								8) Bcl-2 level	9) *p* < 0.01
								9) Bax level	10) *p* < 0.01
								10) Apoptotic cells	—

SD rats: Sprague-Dawley rats. MCAO: middle cerebral artery occlusion. SOD: superoxide dismute. HIF: hypoxia-inducible factor. NFS: neural functional deficit score. IV: infarct volume. ROS: reactive oxygen species. MAO: monoamine oxidase. HVA: homovanillic acid. DOPAC: dihydroxyphenylacetic acid. DA: dopamine. GFAP: glial fibrillary acidic protein. FGF2: Fibroblast growth factor-2. FGFR1: Fibroblast growth factor receptor 1. CDK4: Cyclin-dependent kinase 4. PBS: phosphate-buffered saline.

Nine studies (Shi et al., 2012; Yu et al., 2014; Han et al., 2015; Chen et al., 2016; Liu et al., 2018; Zhang et al., 2018; Zuo et al., 2018; Zhang et al., 2019; Li et al., 2020) adopted IV as outcome measurements; fourteen studies (Chen et al., 2009; Shi et al., 2012; Chen et al., 2014; Yu et al., 2014; Han et al., 2015; Atochin et al., 2016; Liu et al., 2018; Zhang et al., 2018; Zuo et al., 2018; Zhang et al., 2019; Zhong et al., 2019; Li et al., 2020; Zhang et al., 2020; Dong et al., 2021) used NFS as outcome measurements, among them eight studies (Shi et al., 2012; Yu et al., 2014; Han et al., 2015; Liu et al., 2018; Zhang et al., 2018; Zuo et al., 2018; Zhang et al., 2019; Li et al., 2020) adopted both above two outcome measurements. However, the methods used to identify IV were different; eight studies (Shi et al., 2012; Yu et al., 2014; Han et al., 2015; Chen et al., 2016; Liu et al., 2018; Zhang et al., 2018; Zuo et al., 2018; Li et al., 2020) used TTC staining and one study (Zhang et al., 2019) used MRI scan. The standards of NFS were diverse: seven studies (Chen et al., 2009; Shi et al., 2012; Chen et al., 2014; Han et al., 2015; Zhang et al., 2018; Zhang et al., 2019; Li et al., 2020) adopted Zea Longa (ZL) score; one study (Atochin et al., 2016) used McGraw scale; one studies (Yu et al., 2014) used modified neurological severity score (mNSS); 1study (Zuo et al., 2018) used Ludmila Belayev test, For grid test, Beam walk test, and Wire grip test; one study (Zhong et al., 2019) used Balance beam test, foot fault test and mNSS; one study (Dong et al., 2021) used rotarod tests, one study (Liu et al., 2018) used rotarod tests and mNSS, and one study (Zhang et al., 2020) used the method described by Brambrink et al. (2006). The included studies also reported TUNEL-positive cells, Caspase-3, Bcl-2, TNF-α, IL-1, IL-2, IL-6, IL-8, malondialdehyde (MDA), superoxide dismutase (SOD), glutathione (GSH), glutathione-S-transferase (GST), Evans blue content, MBP, MAP2, MAO, ROS, LDH, GLUT3, p53, GFAP, DA, HVA, DOPAC, cyclin D1, CDK4, p27Kip1, cell viability rate, and apoptotic cells.

### Study Quality

The quality of the 15 included studies was evaluated and ranged from 1/10 to 7/10 with the average of 5.53 in Table 2. Of which, four studies (Zuo et al., 2018; Zhang et al., 2019; Zhang et al., 2020; Dong et al., 2021) obtained seven points, seven studies (Shi et al., 2012; Yu et al., 2014; Han et al., 2015; Chen et al., 2016; Liu et al., 2018; Zhong et al., 2019; Li et al., 2020) obtained six points, one study (Atochin et al., 2016) obtained five points, one studies (Zhang et al., 2018) obtained four points, one studies (Chen et al., 2009) obtained three points, and the remaining one study (Liu et al., 2018) obtained one point. All studies were published in peer-reviewed journals. Twelve studies (Shi et al., 2012; Yu et al., 2014; Han et al., 2015; Atochin et al., 2016; Chen et al., 2016; Liu et al., 2018; Zuo et al., 2018; Zhang et al., 2019; Zhong et al., 2019; Li et al., 2020; Zhang et al., 2020; Dong et al., 2021) described control of the room temperature. Ten studies (Chen et al., 2009; Shi et al., 2012; Yu et al., 2014; Chen et al., 2016; Liu et al., 2018; Zuo et al., 2018; Zhang et al., 2019; Li et al., 2020; Zhang et al., 2020; Dong et al., 2021) declared that they had random allocation to treatment and control groups. Twelve studies (Chen et al., 2009; Shi et al., 2012; Han et al., 2015; Atochin et al., 2016; Chen et al., 2016; Zhang et al., 2018; Zuo et al., 2018; Zhang et al., 2019; Zhong et al., 2019; Li et al., 2020; Zhang et al., 2020; Dong et al., 2021) used anesthetic without significant intrinsic vascular protection activity. Thirteen studies (Shi et al., 2012; Yu et al., 2014; Han et al., 2015; Atochin et al., 2016; Chen et al., 2016; Liu et al., 2018; Zhang et al., 2018; Zuo et al., 2018; Zhang et al., 2019; Zhong et al., 2019; Li et al., 2020; Zhang et al., 2020; Dong et al., 2021) mentioned compliance with animal welfare regulations. Nine studies (Shi et al., 2012; Yu et al., 2014; Han et al., 2015; Liu et al., 2018; Zuo et al., 2018; Zhang et al., 2019; Zhong et al., 2019; Zhang et al., 2020; Dong et al., 2021) declared that the model establishment and outcome assessment were conducted in double-blind trial. Twelve studies (Yu et al., 2014; Han et al., 2015; Atochin et al., 2016; Chen et al., 2016; Liu et al., 2018; Zhang et al., 2018; Zuo et al., 2018; Zhang et al., 2019; Zhong et al., 2019; Li et al., 2020; Zhang et al., 2020; Dong et al., 2021) contained statements on potential conflict of interests. There was no study calculating sample size in the animal experiment and blinded assessment of outcome. No study used animals with relevant comorbidities.

**TABLE 2:** Quality assessment of included studies.

Study (years)	1	2	3	4	5	6	7	8	9	10	Total
Atochin et al. (2016)	√	√	—	—	—	√	—	—	√	√	5
Chen et al. (2015)	√	√	√	—	—	√	—	—	√	√	6
Han et al. (2015)	√	√	—	—	√	√	—	—	√	√	6
Liu et al. (2018)	√	√	√	—	√	—	—	—	√	√	6
Shi et al. (2012)	√	√	√	—	√	√	—	—	√		6
Zhang et al. (2018)	√	—	—	—	—	√	—	—	√	√	4
Zhang et al. (2019)	√	√	√	—	√	√	—	—	√	√	7
Yu et al. (2014)	√	√	√	—	√	—	—	—	√	√	6
Zuo et al. (2018)	√	√	√	—	√	√	—	—	√	√	7
Chen et al. (2014)	√	—	—	—	—	—	—	—	—	—	1
Chen et al. (2009)	√	—	√	—	—	√	—	—	—	—	3
Zhong et al. (2019)	√	√	—	—	√	√	—	—	√	√	6
Zhang et al. (2020)	√	√	√	—	√	√	—	—	√	√	7
Dong et al. (2021)	√	√	√	—	√	√	—	—	√	√	7
Li et al. (2020)	√	√	√	—		√	—	—	√	√	6

1: peer-reviewed publication; 2: statements describing control of temperature; 3: randomization to treatment group; 4: allocation concealment; 5: blinded assessment of outcome; 6: avoidance of anesthetics with known notable intrinsic neuroprotective properties; 7: use of animals with relevant comorbidities; 8: sample size calculation; 9: compliance with animal welfare regulations; 10: declared any potential conflict of interest; NR: not reported. HO-1: heme oxygenase-1.

### Effectiveness

#### IV

**FIGURE 2:**
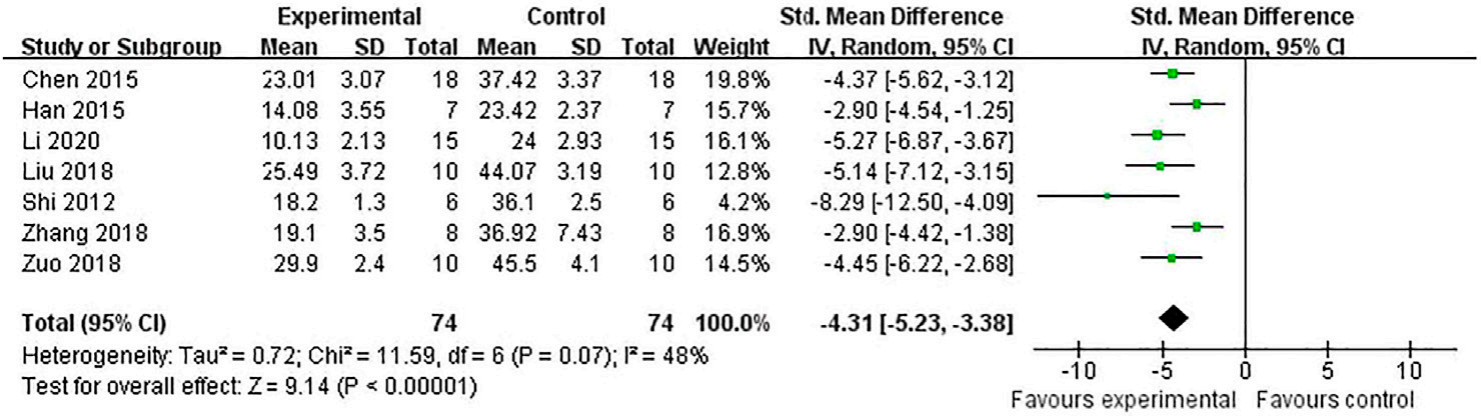
The pooled estimate of RRC for improving infarct volume according to TTC staining.

The IV was measured in nine studies (Chen et al., 2016; Han et al., 2015; Liu et al., 2018; Shi et al., 2012; Zhang et al., 2018; Zhang et al., 2019; Yu et al., 2014; Zuo et al., 2018; Li et al., 2020). Meta-analysis of seven studies (Chen et al., 2016; Han et al., 2015; Liu et al., 2018; Shi et al., 2012; Zhang et al., 2018; Zuo et al., 2018; Li et al., 2020) showed RRC were significant for reducing IV compared with control groups in TTC staining [n = 148, SMD = −4.31, 95% CI (−5.23 to −3.38), *p* < 0.00001; heterogeneity: *χ* ^*2*^ = 11.59, df = 6 (*p* = 0.07), *I* ^*2*^ = 48%] (Figure 2) and one study (Zhang et al., 2019) showed a beneficial effect of RRC for reducing IV according to MRI scans. (*p* < 0.01). One study (Yu et al., 2014) reported that RRC significantly reduced IV.

#### Brain Edema

**FIGURE 3:**
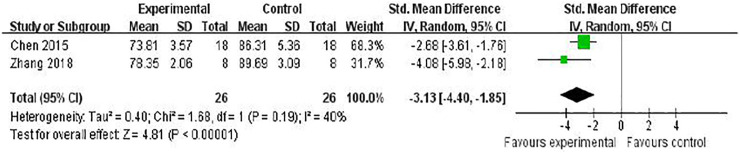
The pooled estimate of RRC for improving brain water content.

Two studies (Chen et al., 2016; Zhang et al., 2018) investigated the effect of RRC on reducing brain edema following MCAO by testing brain water content. Meta-analysis showed a significant reduction [*n* = 52, SMD = −3.13, 95% (CI−4.40 to −1.85), *p* < 0.00001; heterogeneity: *χ* ^*2*^ = 1.68, df = 1 (*p* = 0.19), *I* ^*2*^ = 40%] (Figure 3).

#### NFS

**FIGURE 4:**
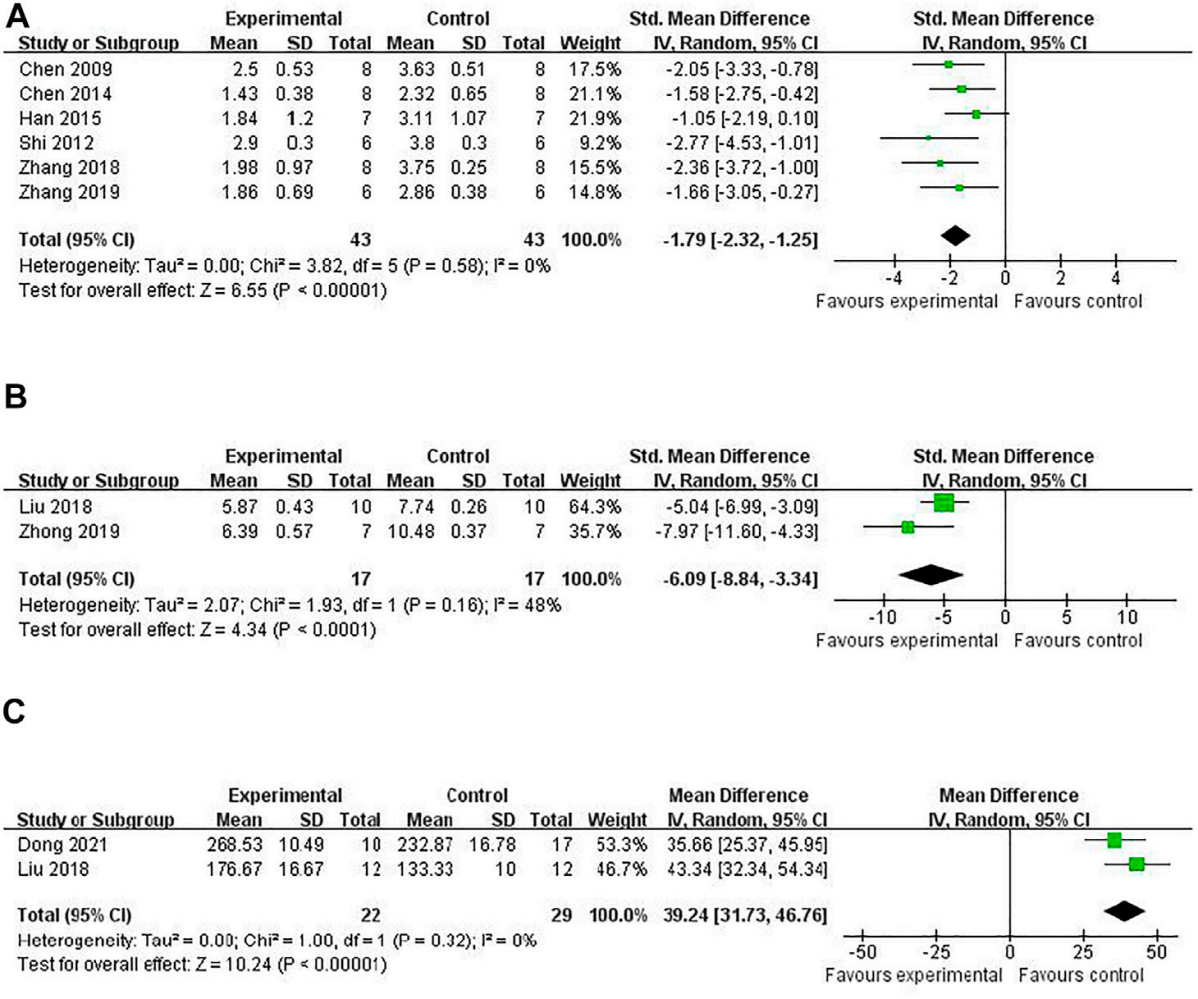
The pooled estimate of RRC for improving neurological function score according to: **(A)** ZL score; **(B)** mNSS; **(C)** Rotarod tests.

The NFS was conducted in 14 studies (Atochin et al., 2016; Han et al., 2015; Liu et al., 2018; Shi et al., 2012; Zhang et al., 2018; Zhang et al., 2019; Yu et al., 2014; Zuo et al., 2018; Chen et al., 2014; Chen et al., 2009; Zhong et al., 2019; Zhang et al., 2020; Dong et al., 2021; Li et al., 2020). Meta-analysis of 6 (Han et al., 2015; Shi et al., 2012; Zhang et al., 2018; Zhang et al., 2019; 2018; Chen et al., 2009; Chen et al., 2014) studies showed that RRC was significant for improving ZL scores compared with the control [(*n* = 86, SMD -1.79, 95% CI (−2.32 to −1.25), *p* < 0.00001; heterogeneity: *χ* ^*2*^ = 3.82, df = 5 (*p* = 0.58), *I* ^*2*^ = 0%] (Figure 4A). Two studies reported that RRC reduced neurologic deficit score of ZL (Yu et al., 2014) and mNSS (Li et al., 2020). Meta-analysis of two studies (Liu et al., 2018; Zhong et al., 2019) showed a significant difference for improving mNSS [*n* = 34, SMD −6.09, 95% CI (−8.84 to −3.34), *p* < 0.0001; heterogeneity: *χ* ^*2*^ = 1.93, df = 1 (*p* = 0.16), *I* ^*2*^ = 48%] (Figure 4B). Meta-analysis of two studies (Liu et al., 2018; Dong et al., 2021) showed a significant difference for increasing the latency to fall off the rotarod in the rotarod test. [*n* = 51, SMD 39.24, 95% CI (31.73 to 46.76), *p* < 0.00001; heterogeneity: *χ* ^*2*^ = 1.00, df = 1 (*p* = 0.32), *I* ^*2*^ = 0%] (Figure 4C).

#### Others

One study (Atochin et al., 2016) found that RRC could improve neurological deficit in McGraw scale. One study (Zuo et al., 2018) showed that RRC improved neurological deficits in Ludmila Belayev test, For grid test, Beam walk test, and Wire grip test compared with the control.

#### Neuroprotective Mechanisms of RRC

**FIGURE 5:**
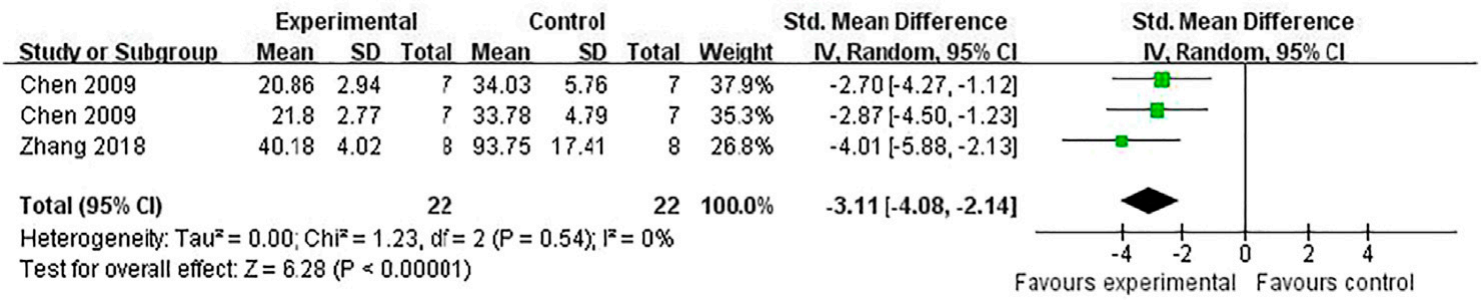
The pooled estimate of RRC for decreasing TUNEL-positive cells.

**FIGURE 6:**
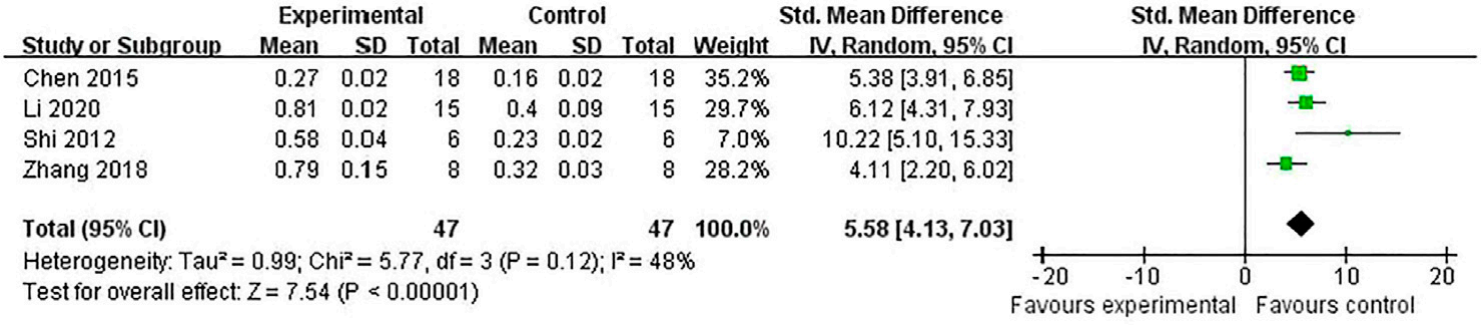
The pooled estimate of RRC for increasing Bcl-2 levels.

**FIGURE 7:**
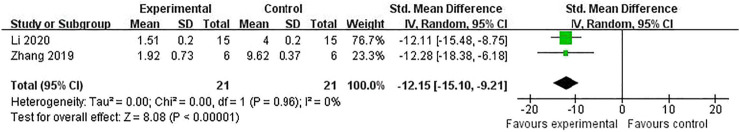
The pooled estimate of RRC for decreasing TNF-α.

**FIGURE 8:**
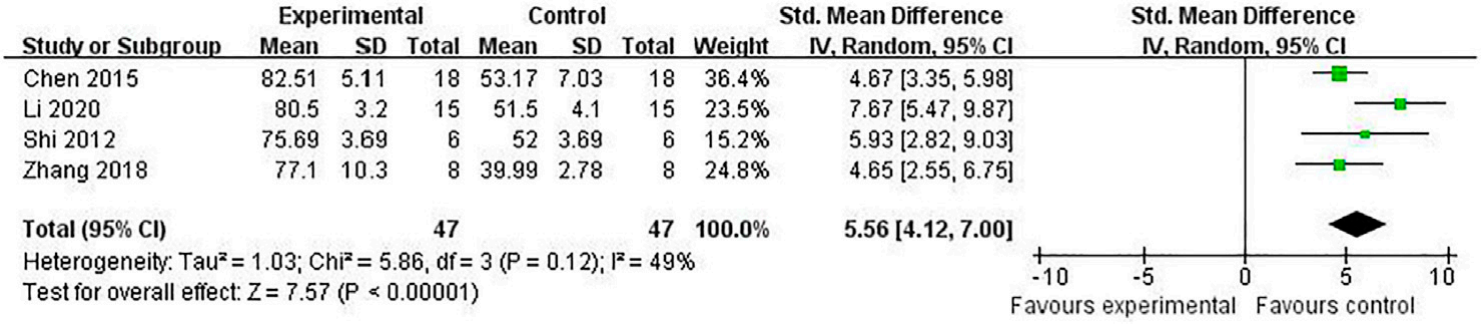
The pooled estimate of RRC for increasing cell viability.

**FIGURE 9:**
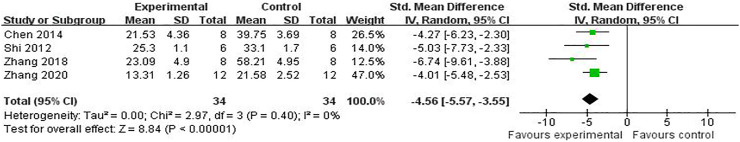
The pooled estimate of RRC for decreasing apoptosis rate.

Compared with controls, meta-analysis of two studies (Zhang et al., 2018; Chen et al., 2009) with three comparisons showed a significant reduction of TUNEL-positive cells [(*n* = 44, SMD = -3.11, 95% (CI −4.08 to −2.14), *p* < 0.00001; heterogeneity: *χ* ^*2*^ = 1.23, df = 2 (*p* = 0.54), *I* ^*2*^ = 0%] (Figure 5). Meta-analysis of four studies (Chen et al., 2016; Shi et al., 2012; Zhang et al., 2018; Li et al., 2020) for reducing Bcl-2 levels [*n* = 94, SMD = 5.58, 95% CI (4.13 to 7.03), *p* < 0.00001; heterogeneity: *χ* ^*2*^ = 5.77, df = 3 (*p* = 0.12), *I* ^*2*^ = 48%] (Figure 6). Meta-analysis of two studies (Zhang et al., 2019; Li et al., 2020) reducing the level of TNF-α [*n* = 42, SMD = −12.15, 95% CI (−15.10 to −9.21), *p* < 0.00001; heterogeneity: *χ* ^*2*^ = 0.00, df = 1 (*p* = 0.96), *I* ^*2*^ = 0%] (Figure 7). Meta-analysis of four studies (Chen et al., 2016; Shi et al., 2012; Zhang et al., 2018; Li et al., 2020) increasing cell viability [*n* = 94, SMD = 5.56, 95% CI (4.12 to 7.00), *p* < 0.00001; heterogeneity: *χ* ^*2*^ = 5.86, df = 3 (*p* = 0.12), *I* ^*2*^ = 49%] (Figure 8). Meta-analysis of four studies (Shi et al., 2012; Chen et al., 2014; Zhang et al., 2018; Zhang et al., 2020) reducing apoptosis rate [*n* = 68, SMD = -4.56, 95% CI ( −5.57 to −3.55], *p* < 0.00001; heterogeneity: *χ* ^*2*^ = 2.97, df = 3 (*p* = 0.40), *I* ^*2*^ = 0%] (Figure 9).

**FIGURE 10:**
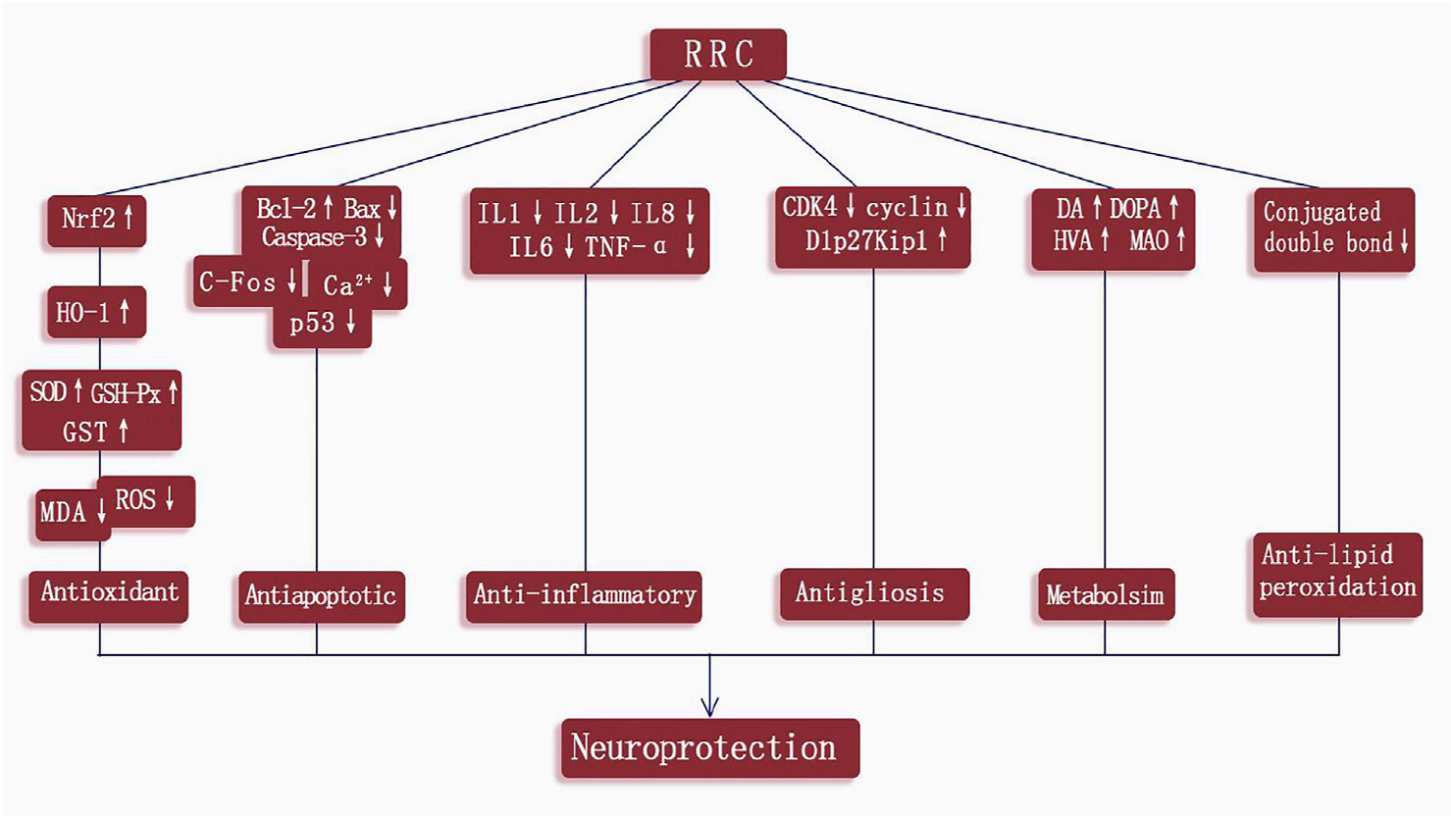
A schematic representation of neuroprotection mechanisms of RRC for ischemic cerebral injury.

According to the included studies, the possible neuroprotective mechanisms of RRC for ischemic stroke lie in the following aspects: 1) RRC could help alleviate the pathological BBB damage (Zuo et al., 2018). 2) RRC could repress lipid peroxidation (Atochin et al., 2016). 3) RRC could effectively reduce oxidative reactions through increasing the activity of SOD, GSH-Px HO-1, Nrf2 and GST and decreasing the concentration of MDA and ROS (Shi et al., 2012; Han et al., 2015). 4) RRC could inhibit the occurrence of inflammation by decreasing the expression of proinflammatory cytokines such as TNF-α, IL-1β, IL-1, IL-2 and IL-6 (Chen et al., 2016; Liu et al., 2018; Zhang et al., 2019; Li et al., 2020). 5) RRC could exert antiapoptotic effects by increasing the levels of Bcl-2 (Shi et al., 2012; Chen et al., 2016; Zhang et al., 2018; Li et al., 2020), decreasing the levels of Bax (Shi et al., 2012; Li et al., 2020), caspase 3 (Li et al., 2020), C-Fos (Chen et al., 2009), GFAP (Liu et al., 2018), p53 (Zhang et al., 2020), decreasing the activity of LDH (Zhang et al., 2018) and reducing TUNEL positive cells (Yu et al., 2014; Liu et al., 2018; Zhang et al., 2018). 6) RRC could exert the neuroprotective effect via regulating BDNK mediated PI3K/Akt pathway (Zhang et al., 2018; Zuo et al., 2018), through calpain1/PKA/CREB pathway (Yu et al., 2014) and through modulating monoamine metabolism (Zhong et al., 2019). 7) RRC could inhibit reactive astrogliosis and glial scar formation, probably through Akt/GSK-3β pathway. Characteristics of mechanism studies of RRC on experimental ischemic stroke were showed in Table 3 and Figure 10.

**TABLE 3:** Characteristics of mechanism studies of RRC on cognition impairment.

Study (years)	Model	Method of administration (experimental group versus control group)	Observations	Possible mechanisms
Atochin et al. (2016)	MCAO (2 h)	P-tyrosol versus normal saline	Attenuated NFS	Repression of lipid peroxidation
			Decreased neurons loss	
			Decreased conjugated content	
Chen et al., 2015	MCAO	Salidroside versus nr	Reduced IV	Repression of inflammatory reactions Inhibition of apoptosis
			Decreased brain water content	
			Increased cell survival rate	
			Decreased TNF-α, IL-1β and IL-6 contents	
			Increased Bcl-2 expression	
Han et al. (2015)	MCAO (2 h)	Salidroside versus normal saline	Reduced IV Attenuated NFS Decreased MDA content Increased SOD, GSH-Px and GST activity Increased Nrf2 and HO-1 expression	Reduction of oxidative reactions Nrf2/antioxidant response element pathway
Liu et al. (2018)	MCAO (1 h)	Salidroside versus normal saline	Reduced IV Attenuated NFS Decreased IL-1β, IL-2, IL-6, IL-8 and TNF-α content Increased MBP expression Increased MAP2 expression	Repression of inflammatory reactions
Shi et al. (2012)	MCAO (2 h)	Salidroside versus normal saline	Reduced IV	Inhibition of apoptosis Reduction of oxidative reactions
			Attenuated NFS	
			Increased cell viability rate	
			Reduced apoptotic cells	
			Decreased ROS level	
			Increased Bcl-2 expression	
			Reduced Bax expression	
Zhang et al. (2018)	MCAO (2 h)	Salidroside versus normal saline	Reduced IV	Inhibition of apoptosis BDNK -mediated PI3K/Akt Pathway
			Attenuated NFS	
			Increased cell viability rate	
			Reduced brain water content	
			Reduced apoptotic cells	
			Reduced TUNEL positive cells count	
			Reduced LDH activity	
			Increased Bcl-2 expression	
Zhang et al. (2019)	MCAO (2 h)	Salidroside versus normal saline	Reduced IV Attenuated NFS Increased NeuN protein level Reduced TNF-α and IL-6 expression	Repression of inflammatory reactions
Yu et al. (2014)	MCAO (2 h)	Salidroside versus normal saline	Reduced IV	Inhibition of apoptosis Calpain1/PKA/CREB pathway
			Attenuated NFS	
			Increased cell viability rate	
			Reduced apoptotic cells	
			Reduced TUNEL positive cells count	
			Increased glucose uptake	
			Increased GLUT3 expression	
			Increased pS133-CREB level	
			Increased PKA RII level	
			Reduced intracellular Ca2+ influx	
Zuo et al. (2018)	MCAO (3 h)	Salidroside versus normal saline	Reduced IV	Activating PI3K/Akt signaling by phosphorylating Akt on Ser473
			Attenuated NFS	
			Reduced Evan’s blue leakage	
			Increased cell viability	
Chen et al. (2014)	MCAO (2 h)	Rhodiola rosea versus normal saline	Attenuated NFS	Inhibition of apoptosis
			Reduced cells apoptotic	
			Reduced C-Fos expression	
Chen et al. (2009)	MCAO (2 h)	Rhodiola rosea versus normal saline	Attenuated NFS Reduced TUNEL positive cells count Reduced GFAP expression	Inhibition of apoptosis
Zhong et al. (2019)	MCAO (2 h)	Salidroside versus normal saline	Attenuated NFS	Modulation of monoamine metabolism
			Increased DA, DOPAC and HVA level	
			Increased MAO level	
Zhang et al. (2020)	MCAO (8min)	Salidroside versus Distilled water	Attenuated NFS	Inhibition of apoptosis
			Reduced p53 level	
			Reduced apoptotic cells	
			Increased Bcl-2 expression	
			Reduced Bax expression	
Dong et al. (2021)	MCAO (1 h)	Salidroside versus normal saline	Attenuated NFS	Inhibits reactive astrogliosis and glial scar formation Akt/GSK-3β Pathway
			Reduced GFAP expression	
			Reduced cyclin D1 expression	
			Reduced CDK4 expression	
			Increased p27Kip1 level	
Li et al. (2020)	MCAO (2 h)	Salidroside versus normal saline	Attenuated NFS	Repression of inflammatory reactions Inhibition of apoptosis
			Reduced IV	
			Increased FGF2/FGFR1 expression	
			Reduced TNF-α, IL-1β and IL-6 expression	
			Increased Bcl-2 level	
			Decreased caspase 3 level	
			Decreased Bax level	
			Increased cell viability rate	
			Inhibits neuron apoptotic	

SD rats: Sprague-Dawley rats. MCAO: middle cerebral artery occlusion. SOD: superoxide dismute. HIF: hypoxia-inducible factor. NFS: neural functional deficit score. IV: infarct volume. ROS: reactive oxygen species. MAO: monoamine oxidase. HVA: homovanillic acid. DOPAC: dihydroxyphenylacetic acid. DA: dopamine. GFAP: glial fibrillary acidic protein. FGF2: Fibroblast growth factor-2. FGFR1: Fibroblast growth factor receptor 1. CDK4: Cyclin-dependent kinase 4.

## Discussion

### Summary of Results

To our knowledge, it is the first preclinical systematic review to assess the efficacy of RRC for cerebral ischemic stroke. In the present study, 15 studies with 345 animals showed that RRC significantly improved NFS and reduced IV in cerebral ischemia animal models. Thus, RRC exerted the potential neuroprotective function for ischemic stroke, mainly through anti-inflammatory, anti-apoptosis, and anti-oxidative and alleviating the pathological BBB damage. However, given methodological weaknesses, the overall available evidence from the present study should be interpreted cautiously. Thus, the conclusions in the present study should be partially treated with caution.

### Limitations

There are several limitations in the primary studies. Firstly, only Chinese and English literatures were searched, which may cause selection bias as studies published in other languages were absent (Zhang et al., 2019). Secondly, no study had used an animal with co-morbidities, such as hypertension, diabetes or hyperlipidemia (Heusch, 2017), which would be more relevant models for human pathology (Guyatt et al., 2011). Thirdly, the studies had methodological deficiencies. None of these studies reported the blindness of ischemia induction, allocation concealment, randomization to treatment group or control group and sample size calculation, which are the core criteria of study design. Thus the analysis may result in overestimated effect size (Ospina et al., 2005; Higgins and Green, 2012). Thereby, the results in the present study should be interpreted with caution.

### Implications

The damage inflicted on the neuron during ischemic stroke is a complex process, involving multiple factors. The main mechanisms of injury are oxidative and nitrative stress, inflammation, apoptosis, ion imbalance, calcium overload, and energy depletion (Terasaki et al., 2014; Jayaraj et al., 2019), leading to neurovascular unit dysfunction and neurologic deficitse. Thus, neuroprotective drugs generally work through one or combined aspects of the above targets. The present study showed RRC could exert potential neuroprotective effects in experimental for ischemic stroke indicating that RRC are candidates for ischemic stroke treatment and can be used for further clinical trials. The possible mechanisms of RRC for cerebral ischemia injury are summarized as follows: 1) alleviating the pathological BBB damage; 2) repressing lipid peroxidation; 3) antioxidant through increasing the activity of SOD, HO-1, Nrf2, GSH-Px and GST and decreasing the concentration of MDA and ROS; 4) anti-inflammatory via decreasing the expression of proinflammatory cytokines such as TNF-α, IL-1β, IL-1, IL-2 and IL-6; 5) anti-apoptotic via increasing the levels of Bcl-2, decreasing the levels of Bax, caspase3, C-Fos, GFAP, p53, decreasing the activity of LDH and reducing TUNEL positive cells; 6) neuroprotective effect via regulating BDNK-mediated PI3K/Akt pathway, through calpain1/PKA/CREB pathway and through modulating monoamine metabolism; 7) inhibiting reactive astrogliosis and glial scar formation, probably through Akt/GSK-3β pathway. To summarize, the possible mechanisms of RRC for ischemic stroke are through antioxidant, lipid peroxidation, anti-apoptosis, anti-inflammatory, improving blood vessel endothelium differentiation, and cerebral metabolism. A recent review (Sun et al., 2020) illustrated that *Rhodiola rosea L.* and its components, particularly salidroside has strong antioxidant activity through regulating mitochondrial biogenesis, repressing ROS production, increasing the activity of the antioxidant enzymes (such as GSH-Px and SOD), and via various signaling pathways (AMPK, PI3K/Akt, Mitochondria-dependent, Nrf2). In addition, another review (Pu et al., 2020) showed that *Rhodiola rosea L.* and its compounds have immune-regulation effects through some inflammatory mediators, such as IL-6, TNFα, IL-1β, and NO, and signaling pathways, such as NF-κ B, AP-1, and STAT3. In the present study, the mechanisms are consistent with the evidences.

Preclinical animal research plays a critical role in human diseases understanding (Murphy and Murphy, 2010). However, original preclinical research is often conducted with a poor methodological quality, which is considered as a hindrance to the translation of animal research into effective preclinical drug treatments for human disease (Baginskait, 2012; Moher et al., 2015). The systematic review can dentify defects in study design, integrate preclinical evidence and guide potential clinical translation (Macleod et al., 2005; De Vries et al., 2014). In the present analysis, the average CAMARADES score of the included studies ranged from 1/10 to 7/10. The main flaws are lacking of sample size calculation, poor blinding in model induction and outcome assessment. Inadequate sample size can miss the real intervention effect in an experiment, while excessive sample size will result in wasting resources and raising animal ethical issues (Arifin and Zahiruddin, 2017; Chen et al., 2019). Poor blinding in outcome assessment could result in a 27% overestimation of the mean reported effect size (Holman et al., 2015) Additionally, all the animal experiments are conducted in healthy animals which lack the comorbidities, such as diabetes, hypertension and hyperlipidemia. Reporting guidelines set detailed predetermined standards to make biomedical research report more complete and transparent, and enhancing their value in scientific exploration and clinical practice. The Animal Research: Reporting of *In Vivo* Experiments (ARRIVE) (Kilkenny et al., 2012) is a reporting guidelines, which are organized into twenty sections, providing recommendations on Introduction, Methods, Results, and Discussion. The ARRIVE guidelines were recommended to be utilized when designing and reporting animal research on RRC for ischemic stroke which can provide guidance on the complete and transparent reporting of *in vivo* animal researches, helping to improve the quality of further researches (Karp et al., 2015). Thus, we suggest that further animal researches should follow up the reporting guidelines, increasing the value of clinical trials and further application. Furthermore, the following factors need to be considered: 1) method by which sample size was determined should be appropriately detailed; 2) experimental animals have relevant comorbidities, which are like human pathology under the clinical conditions; 3) primary outcome should be closer to clinical practice.

## Conclusion

This study showed that RRC exerted potential neuroprotective effects in ischemic stroke largely through anti-oxidative, anti-inflammatory, antigliosis, anti-apoptotic, neuroprotective, and alleviating the pathological BBB damage mechanisms. In addition, this systematic review provides an experimental evidence-based suggestion that RRC may be a promising candidate for clinical trials.

## Data Availability

The original contributions presented in the study are included in the article/Supplementary Material, further inquiries can be directed to the corresponding authors.
